# Inhibition of the Proprotein Convertases Represses the Invasiveness of Human Primary Melanoma Cells with Altered *p53*, *CDKN2A* and *N-Ras* Genes

**DOI:** 10.1371/journal.pone.0009992

**Published:** 2010-04-09

**Authors:** Claude Lalou, Nathalie Scamuffa, Samia Mourah, Francois Plassa, Marie-Pierre Podgorniak, Nadem Soufir, Nicolas Dumaz, Fabien Calvo, Nicole Basset-Seguin, Abdel-Majid Khatib

**Affiliations:** 1 INSERM, UMRS940, Equipe Avenir, Institut de Génétique Moléculaire, Hôpital Saint-Louis, Université Paris 7, Paris, France; 2 Laboratoire de Biochimie, Hôpital Saint-Louis, Paris, France; 3 Laboratoire de Biochimie Hormonale et Génétique, Hôpital Bichat, Paris, France; 4 INSERM U976, Hôpital Saint-Louis, Paris, France; City of Hope National Medical Center, United States of America

## Abstract

**Background:**

Altered tumor suppressor *p53* and/or *CDKN2A* as well as *Ras* genes are frequently found in primary and metastatic melanomas. These alterations were found to be responsible for acquisition of invasive and metastatic potential through their defective regulatory control of metalloproteinases and urokinase genes.

**Methodology/Principal Findings:**

Using primary human melanoma M10 cells with altered *p53*, *CDKN2A* and *N-Ras* genes, we found that inhibition of the proprotein convertases (PCs), enzymes involved in the proteolytic activation of various cancer-related protein precursors resulted in significantly reduced invasiveness. Analysis of M10 cells and their gastric and lymph node derived metastatic cells revealed the presence of all the PCs found in the secretory pathway. Expression of the general PCs inhibitor α1-PDX in these cells in a stable manner (M10/PDX) had no effect on the mRNA expression levels of these PCs. Whereas, *in vitro* digestion assays and cell transfection experiments, revealed that M10/PDX cells display reduced PCs activity and are unable to process the PCs substrates proIGF-1R and proPDGF-A. These cells showed reduced migration and invasion that paralleled decreased gelatinase MMP-2 activity and increased expression and secretion of tissue inhibitor of metalloproteinase-1 (TIMP-1) and TIMP-2. Furthermore, these cells showed decreased levels of urokinase-type plasminogen activator receptor (uPAR) and increased levels of plasminogen activator inhibitor-1 (PAI-1).

**Conclusions:**

Taken together, these data suggest that inhibition of PCs activity results in decreased invasiveness of primary human melanoma cells despite their altered *p53*, *CDKN2A* and *N-Ras* genes, suggesting that PCs may serve as novel therapeutic targets in melanoma.

## Introduction

Frequently arising from precursor lesions know as atypical nevi, melanomas constitute one of main causes of morbidity and mortality, although when detected early, many melanomas are curable by surgical intervention [1]. In addition to poor prognosis due to their high tendency to form metastasis, advanced melanomas are also highly resistant to various treatments, including radiation and chemotherapy [2]. Recently, many studies revealed that various genetic changes are usually associated with the progression of the disease. These include the loss or inactivation of tumor suppressor genes such as *CDKN2A* and *p53* [3], [4] and alteration of other genes such as *Ras* [5]. Indeed, the corresponding *CDKN2A* product (p16 protein) was reported to act by blocking progression through the cell cycle [3], [4] and to suppress tumor invasion and metastasis by acting as an inhibitor of expression of various matrix metalloproteinases in tumor cells [6]–[8]. Similarly, *p53* that encodes a multifunctional transcription factor to mediate cellular responses to diverse stimuli, including apoptosis, DNA repair, and cell cycle arrest [9]–[11] was also found to regulate the proteolytic capacity of various cells by directly controlling the expression of various MMPs [12]–[14] and urokinase genes [15]–[17]. Indeed, the promoter of several MMPs was found to contain a consensus *p53* binding element, which mediates activation of transcription of these genes [12]. In addition wild-type and mutant *p53* differentially regulate the activity of the promoters of MMPs, suggesting a direct role for *p53* inactivation in the extra cellular matrix (ECM) degradation, leading to cancer cell invasion [12], [15], [16], [18]. In the same way, altered *Ras* gene was reported to increase various MMPs and urokinase expression as well as tumor cell invasion [19], [20], whereas it's suppression resulted in reduced melanoma cell migration and invasion [21].

A wide range of proteins that control the metastatic character of various tumor cells, including melanoma cells, such as adhesion molecules, growth factors, growth factor receptors and various proteases, are synthesized as inactive precursor proteins that are converted to their bioactive forms directly or indirectly by one or more of the 7 known subtilisin/kexin-like proprotein convertase (PCs) family members. These include PC1, PC2, Furin, PC4, PC5, PACE4, and PC7 [22]–[28]. Previously, altered levels and activity of one or more of these PCs as well as their substrates were reported to be associated with several human cancers, suggesting a direct role of these proteases in the acquisition of the tumorigenic and metastatic potential of tumor cells [22]–[28]. In this study we found that inhibition of PCs in primary human melanoma cells with altered tumor suppressor genes *CDKN2A* and *p53*, and *N-Ras* gene suppress the invasive phenotype by acting on the expression and/or activation of several ECM-degrading enzymes and their inhibitors. These data suggest that proprotein convertases may serve as novel therapeutic targets in the treatment of melanoma.

## Materials and Methods

### Cell culture and transfections

The primary melanoma M10 cells were derived from a 65-years old female patient suffering from primary recurrent malignant melanoma of the leg [29]. The MT10 and MG10 cells were isolated from gastric and lymph node metastatic lesions that arose from primary lesions 3 and 4 years later, respectively [30]. Cells were maintained with RPMI 1640 media containing 10% FCS, 100 U/ml penicillin, and 100 µg/ml streptomycin (Invitrogen, Cergy Pontoise, France). The M10 and Colo829 human melanoma cells were stably transfected with empty pIRES2-EGFP vector or with the same vector containing the full-length cDNA encoding the general PCs inhibitor α1-PDX, as previously described [25], using the Fugen 6.0 (Roche Diagnostics, Meylan, France) according to manufacturer's instructions. After cell transfection, a mixture of G418-resistant (M10/PDX, Colo829/PDX) cells were selected and screened for α1-PDX expression by Western blotting. To generate single-mixture cells expressing the α1-PDX inhibitor, the M10/PDX and Colo829/PDX cells were cultured in the presence of 1 µg/ml *Pseudomonas* exotoxin A [25]. This toxin mediates cell death only after its cleavage by the PCs [25], [31]. In some experiments, cells were transiently transfected with pIRES2-EGFP-V5 empty vector or containing the PCs substrate PDGF-A cDNA [26]. Cells were grown in RPMI-1640 medium supplemented with 10% FCS, 100 U/ml penicillin, 100 mg/ml streptomycin, and 200 µg/ml Geneticin (G418).

### RT-PCR

Following cellular RNA extraction using Trizol reagent (Life Technologies, Inc.) according to the manufacturer's instructions, 2 µg of total RNA were reverse transcribed using a reaction mixture as previously described [25]. The reaction mixture was incubated for 10 min at 25°C, then for 45 min at 42°C, and finally for 5 min at 95°C. The upstream and downstream primers used in this study were previously described [25], [32], [33]. The cDNA amplification was performed using established procedures with slight modifications [25]. Twenty-five cycles consisting of 15 s at 94°C, 15 s at 58°C, and 30 s at 72°C were used, and followed by a 10-min incubation at 72°C. The amplified DNA fragments were analyzed without further purification by electrophoresis on a 1% agarose gel.

### Real-time PCR

One µg of total RNA was subjected to cDNA synthesis using the Superscript II first strand cDNA synthesis system (Invitrogen, Cergy Pontoise, France) and oligo dT_18–20_ as primers. The relative quantification of specific mRNAs was performed by real-time PCR using the StepOnePlus™ Real-Time PCR System and PCR Master Mix (Applied Biosystems, Courtaboeuf, France) according to the manufacturer's instructions. Briefly, the reaction mixture of the reaction (20 µl) contained 2 µl of cDNA resulting of 5-fold dilution of the RT mixture product, 2 x TaqMan Universal PCR Master Mix, 0.3 µM of the probe and 0.9 µM of the forward and reverse primers. PCR reaction was performed at 94°C for 15 s and at 60°C for 1 min during 40 cycles. The transcription of β2-microglobulin evaluated in each sample was used as endogenous control.

### DNA sequencing

Double stranded polymerase chain reactions were carried out on the relevant DNA, and the DNA products were purified by ethanol precipitation and dissolved in 20 µl of sterile double distilled water. Sequencing reactions were then carried out using the Thermo Sequenase Dye Terminator Cycle sequencing premix kit (Amersham,) using standard protocols for the ABI 373 sequencer. Data are presented in chromatogram form where C = blue, A = green, T = red, and G = black.

### Western blotting

Cells were lysed in phosphate-buffered saline (PBS) containing 2% Nonidet P-40 and protease inhibitors (Roche). Media or lysates were subjected to SDS-polyacrylamide gel electrophoresis in 8% gels and proteins were blotted onto nitrocellulose membranes. The primary antibodies used were anti-IGF-I Receptor (Santa Cruz Biotechnology) and anti-V5, for PDGF-A-V5 detection (Invitrogen). Primary antibodies were revealed by horseradish peroxidase-conjugated secondary antibodies (Amersham, Pharmacia Biotech) and Enhanced Chemiluminescence (ECL+Plus, Amersham Pharmacia Biotech) according to the manufacturers' instructions.

### Measurement of TIMP-1, TIMP-2 and uPAR production by tumor cells

Tumor cells were plated in 6-well plates and incubated at 37°C in serum-free media. After 24 hours, media were collected and analyzed for the presence of TIMP-1, TIMP-2 and uPAR using ELISA kit according to the manufacturer's instructions (R&D Systems). Protein concentrations were calculated from a standard curve.

### Measurement of proprotein convertases activity

PCs activity in cells was assessed by evaluating their ability to digest the universal PCs substrate, the fluorogenic peptide pERTKR-MCA, as previously described [33]. In brief, tissue or cell extracts were incubated with pERTKR-MCA (100 µM) during the indicated time periods in the presence of 25 mM Tris, (pH 7.4), 25 mM methyl-ethane-sulfonic acid, and 2.5 mM CaCl_2_, at 37°C and the fluorometric measurements were performed using a spectrofluorometer (FLUOstar OPTIMA; BMG Labtech, Cs/Marne, France).

### Gelatin zymography

Zymography assay was done on serum-free conditioned media derived from control cells or cells stably transfected with α1-PDX [25], [32]. SDS-PAGE gels were copolymerized with gelatin and samples were loaded onto gels without boiling. The gels were washed at room temperature in renaturing solution and in 10 mM of Tris-HCl (pH 8). For the enzymatic reaction to take place, the gels were incubated at 37°C in a solution of 50 mM of Tris-HCl (pH 8) containing 10 mM of CaCl_2_. The gels were then stained in Coomassie blue R250 solution and regions without staining were indicative of gelatin lysis.

### Cell migration and invasion assays

Cell migration and invasion were determined using 24-well microchemotaxis chamber alone or precoated with 2 µg Matrigel (Becton Dickinson Labware) [25], [32]. Control M10 cells or M10 cells expressing α1-PDX cDNA were resuspended in serum-free media and loaded into the upper chamber of each well. Cells were incubated at 37°C for 24 h, after which, the filters were fixed and stained with Diff-Quik (Medion Diagnostic). Cell migration and invasion were quantified by the determination of the number of cells that migrated directly through the membrane toward the medium containing 10% serum that was used as a chemoattractant. Cells detected in each well were counted and the results were represented as (number of migrated cells/number of total cells) x 100%. The human melanoma Colo829 control cells or stably expressing α1-PDX cDNA (Colo829/PDX) were used for comparison in these assays.

## Results

### Altered *p53*, *CDKN2A* and *Ras* genes in the human primary melanoma cells M10

**Figure 1:**
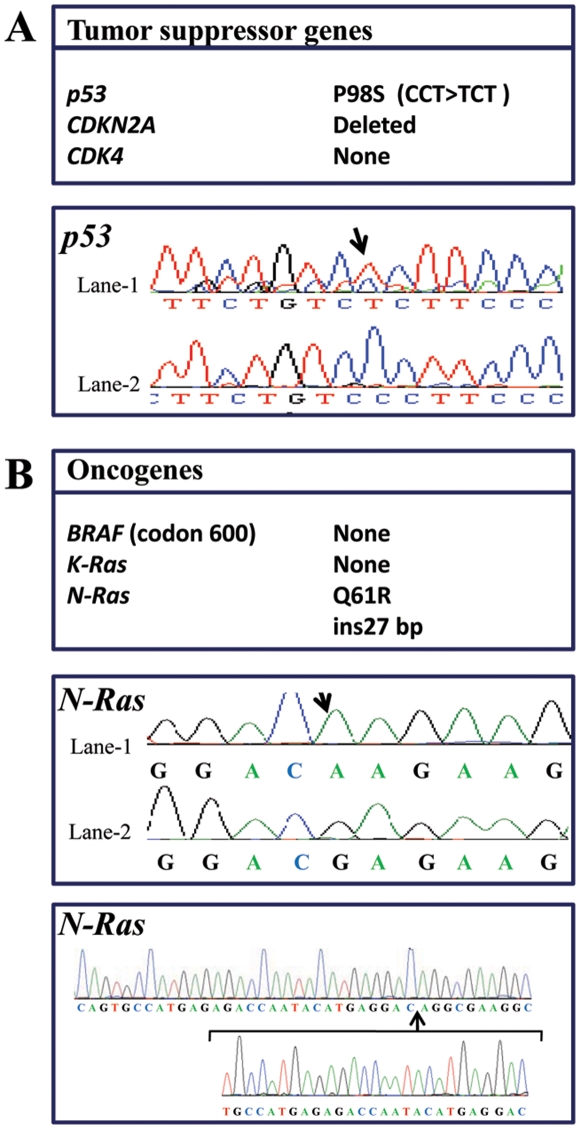
Chromatograms showing the altered genes detected in human primary melanoma M10 cells. (**A**) Sequence analysis of *p53*, *CDKN2A* and *CDK4* genes in M10 cells revealed a P98S substitution in *p53* gene and a *CDKN2A* gene deletion. The CCT>TCT transition in *p53* gene is indicated in Lane-1 and normal chromatogram picture for the relevant section of *p53* gene is shown for comparison in Lane-2. (**B**) Chromatograms of the two *NRas* gene mutations found in M10 cells. Arrows indicate a substitution (CAA>CGA) and an insertion at the 27 bp of the *NRas* gene. In Lane-1, the CAA>CGA substitution in *NRas* gene is indicated, and normal chromatogram for the relevant section of *NRas* gene is shown in Lane-2. The sequence of the 27 bp insert in *NRas* gene is also shown. In these cells no alterations were found in the *BRaf* and *KRas* genes.

Previously, alterations in the tumor suppressor genes *p53* and *CDKN2A* were reported to be critical events in various cancers including melanoma [4]. Similar mutations in various oncogenes such *as Ras* were directly linked to melanoma [5]. Analysis of these genes in the primary human melanoma M10 cells revealed that in *p53* gene, a CCT>TCT transition mutation were found leading to a P98S substitution (Fig. 1A). Whereas analysis of *CDKN2A* gene in these cells revealed its deletion and additional analysis of the oncogenes *CDK4*, *BRAF*, *K-Ras* and *N-Ras* revealed that the latter was altered. Indeed, two mutations were found that include an insertion at the 27 bp and a substitution (CAA>CGA) leading to a Q61R substitution (Fig. 1B). Analysis of the M10 cells-derived metastatic MT10 and MG10 cells revealed the presence of identical gene alterations (data not shown).

### Expression of α1-PDX in human primary melanoma cells

**Figure 2:**
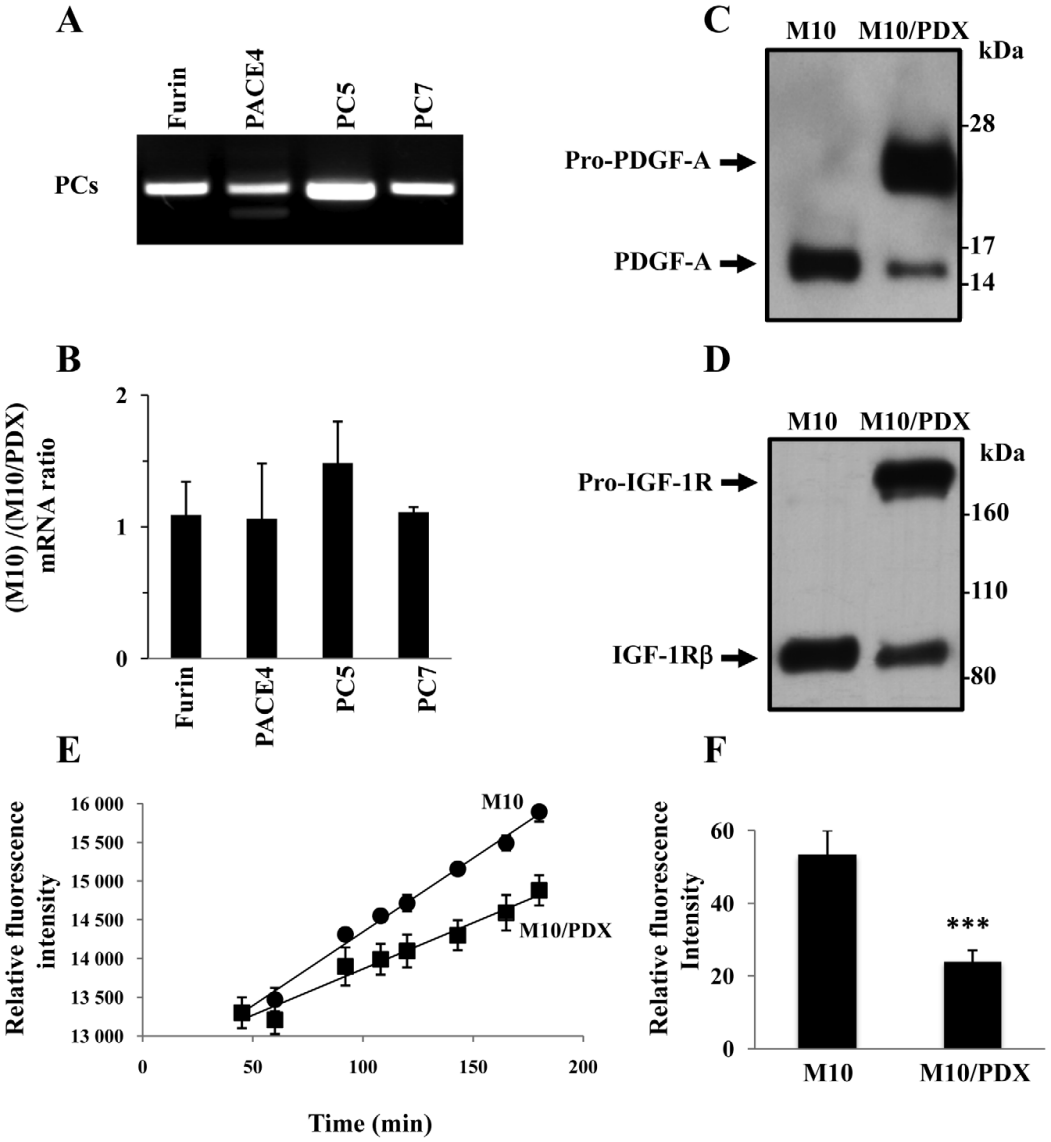
Furin, PACE4, PC5 and PC7 expression and activity in human primary melanoma cells. (**A**) Expression of the indicated PCs was analyzed in M10 cells using specific primers for the PCs found in the secretory pathway (Furin, PACE4, PC5, PC7) and reverse transcription-PCR analysis assay. Note that all these PCs are expressed in the M10 cells. (**B**) Following total RNA extraction from indicated cells, real-time PCR analysis was performed using specific primers for Furin, PACE4, PC5, PC7 as described in Materials and Methods. During PCR, the transcription of β2-microglobulin that was evaluated in each sample was used as endogenous control. Results shown in the bar graph are expressed as ratio of PCs mRNA transcripts (M10)/(M10/PDX) deduced from values derived from M10 and M10/PDX cells mRNA analysis. Data are shown as means ± S.E of three experiments performed in duplicate. Real-time PCR analysis revealed that expression of α1-PDX in M10 cells did not affect significantly the expression levels of these PCs in M10 cells. Processing of proPDGF-A (**C**) and proIGF-IR (**D**) analyzed by Western blotting revealed that expression of α1-PDX in M10 cells (M10/PDX) completely inhibited the processing of pro-IGF-1R and proPDGF-A. (**E**) PCs activity in M10 cells and M10/PDX cells was assessed by evaluating the cell extracts for their ability to digest the universal PCs substrate, the fluorogenic peptide pERTKR-MCA at the indicated time points. Expression of α1-PDX in M10 cells reduced their PCs activity. (**F**) Results shown in the bar graph represent the PCs activity at 2 hours of incubation of the indicated tumor cells. Results are representative of three experiments and data are mean ± S.E performed in triplicate. ***p*<*0.0001.

To investigate the effect of PCs inhibition on the malignant phenotypes of human primary melanoma cells, M10 cells were stably transfected with pIRES2-EGFP empty vector or containing the PCs inhibitor α1-PDX cDNA (M10/PDX). Using RT-PCR analysis, we found that M10 cells express all the PCs found in the secretory pathway namely Furin, PACE4, PC5 and PC7 (Fig. 2A). Similarly, PCR analysis confirmed the presence of empty pIRES2-EGFP vector and pIRES2-EGFP α1-PDX in the transfected cells and that the M10 derived metastatic MT10 and MG10 cells also expressed these PCs (data not shown). In M10/PDX cells, the mRNA levels of the PCs analyzed were not affected significantly following expression of α1-PDX (Fig. 2B).

### PCs activity in M10/PDX cells

To evaluate the effect of α1-PDX on PCs activity in M10 cells, we assessed their ability to convert two known PCs substrates into mature form: PDGF-A tagged with the V5 epitope [26] and insulin-like growth factor-I receptor (IGF-IR) [25]. As shown in Fig. 2C, transfection of M10 cells with PDGF-A cDNA revealed the ability of these cells to process pro-PDGF-A into mature PDGF-A. In contrast, transfection of M10/PDX cells with PDGF-A cDNA resulted in pro-PDGF-A processing blockade. Similarly, analysis of proIGF-IR processing in these cells revealed that M10/PDX cells failed to adequately process proIGF-IR (Fig. 2D). Using an *in vitro* enzymatic digestion assay to assess the ability of tumor cells to digest the universal PCs substrate, the fluorogenic peptide pERTKR-MCA [33], we confirmed the presence of high PCs activity in M10 cells that was inhibited following expression of PCs inhibitor α1-PDX (Fig. 2E, 2F).

### Effect of PCs inhibition on human primary melanoma cells migration and invasion

**Figure 3:**
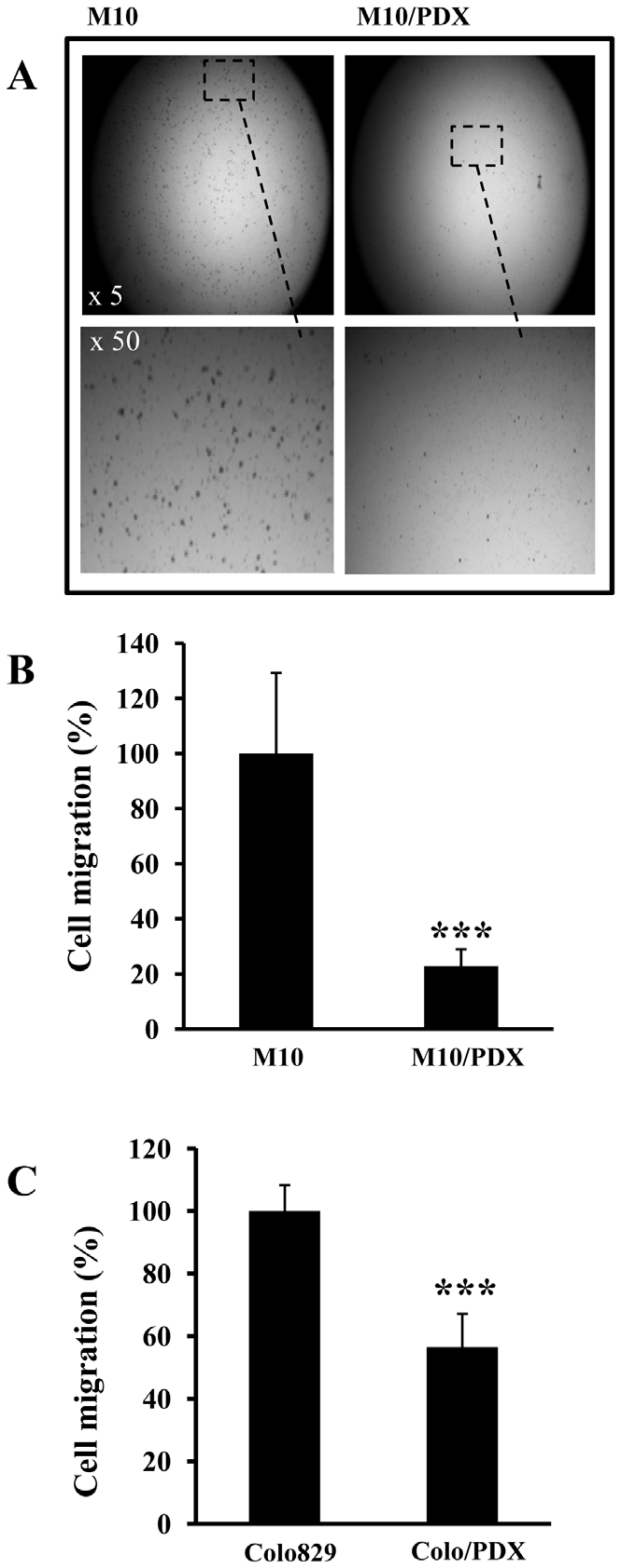
Effect of PCs inhibition on tumor cell migration. Boyden chamber assays were performd using M10 and M10/PDX cells (**A, B**) or Colo829 and Colo829/PDX cells (**C**) to analyze cell migration. Data are shown as means ± S.E of three experiments performed in triplicates and indicate the percentage of the migrating cells. Student's *t* test was used for statistical analysis. ***, *P*<0.001.

**Figure 4:**
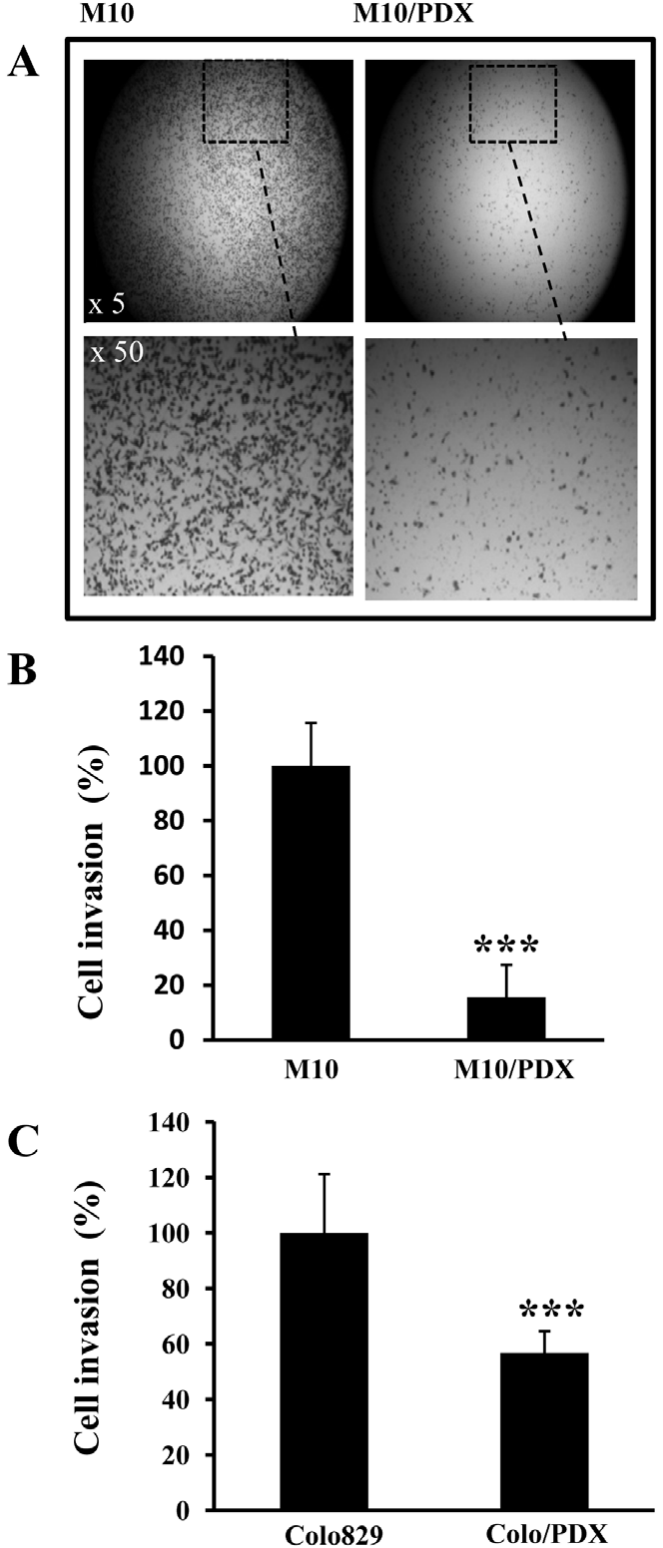
Effect of PCs inhibition on tumor cell invasion. Boyden chamber assays were perfoemed using M10 and M10/PDX cells (**A, B**) or Colo829 and Colo829/PDX cells (**C**) to analyze cell invasion in Matrigel. Data are shown as means ± S.E of three experiments performed in triplicates and indicate the percentage of the migrating cells. Student's *t* test was used for statistical analysis. ***, *P*<0.001.

To evaluate whether inhibition of PCs in primary melanoma cells can affect their migration and invasion, cells were incubated for 24 hours in a microchemotaxis chamber (Fig. 3A, 3B) or chamber pre-coated with Matrigel (Fig. 4A, 4B). As illustrated, the expression of α1-PDX in these cells decreased their ability to migrate and invade. Similarly, stable expression of α1-PDX in melanoma Colo829 cells (Colo829/PDX) resulted in reduced migration (Fig. 3C) and invasion (Fig. 4C).

### Effect of PCs inhibition on gelatinase enzymatic activity and MMP-2/TIMP-1 and MMP-2/TIMP-2 balance

**Figure 5:**
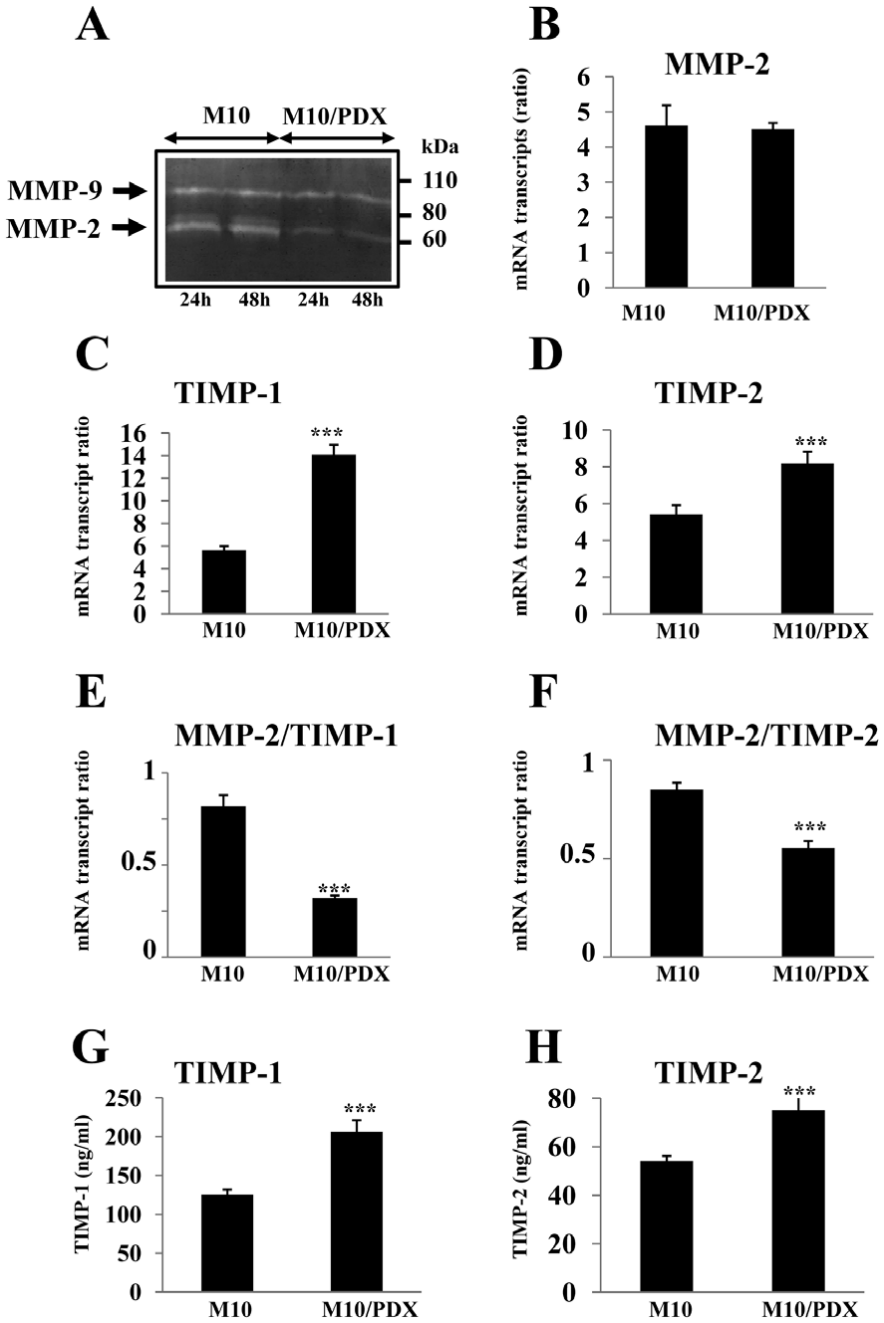
Effect of PCs inhibition on gelatinase activity and MMP-2/TIMP-1 and MMP-2/TIMP-2 ratios. (**A**) Serum-free media derived from M10 and M10/PDX cells were collected and analyzed for gelatinase enzymatic activity as described in Materials and Methods. Following total RNA extraction, real-time PCR analysis was performed using specific primers for MMP-2 (**B**), TIMP-1 (**C**), TIMP-2 (**D**) or β2-microglobulin as described in Materials and Methods. During PCR, the transcription of β2-microglobulin that was evaluated in each sample was used as endogenous control. Results are expressed as mRNA transcript ratios. Data are shown as means ± S.E of three experiments performed in duplicate. (**E, F**) The ratios of MMP-2/TIMP-1 and MMP-2/TIMP-2 are deduced from data obtained in (B), (C) and (D). (**G, H**), Tumor cells were incubated at 37°C in serum-free media. After 24 hours, media were collected and analyzed for the presence of TIMP-1 and TIMP-2 using ELISA kit. Results shown are representative of 3 experiments. Data are mean ± SEM (*n = *6 per group). Student's *t* test was used for statistical analysis ***, *P*<0.001.

Following 24–48 hours of cell incubation under serum-free conditions, media were collected and analyzed for their enzymatic activity. As shown in Fig. 5A, media derived from M10 cells presented high MMP-2 and MMP-9 activity. Expression of α1-PDX in these cells resulted in decreased MMP-2 activity. In contrast, the activity of MMP-9 did not change significantly.

Previously, TIMP-1 and TIMP-2 were described as the major naturally occurring inhibitors of MMP-2, although TIMP-2 was reported to inhibit MMP-2 over 10-fold more effectively than TIMP-1 [34]. To rationalize the observed reduced activity of MMP-2 in M10/PDX cells, we analyzed the expression of MMP-2 and their inhibitors TIMP-1 and TIMP-2 using real-time PCR and ELISA assays. We found that the expression of MMP-2 in these cells wasn't affected by PCs inhibition (Fig. 5B), suggesting that this reduced activity as revealed by the zymography assay is mainly due to post-translational repression. Indeed, in M10/PDX cells mRNA expression level of TIMP-1 and TIMP-2 was significantly increased (Figs. 5C, 5D). Similarly, at the protein level, the amount of TIMP-1 and TIMP-2 collected 24 hours following incubation of cells in serum-free media was increased in cells expressing α1-PDX, as determined by ELISA (Figs. 5G, 5H). Evaluation of MMP-2/TIMP-1 (Fig. 5E) and MMP-2/TIMP-2 (Fig. 5F) ratios indicated that compared to control M10 cells, these ratios were significantly decreased in M10/PDX cells.

### Effect of PCs inhibition on uPA, uPAR and PAI-1 expression

**Figure 6:**
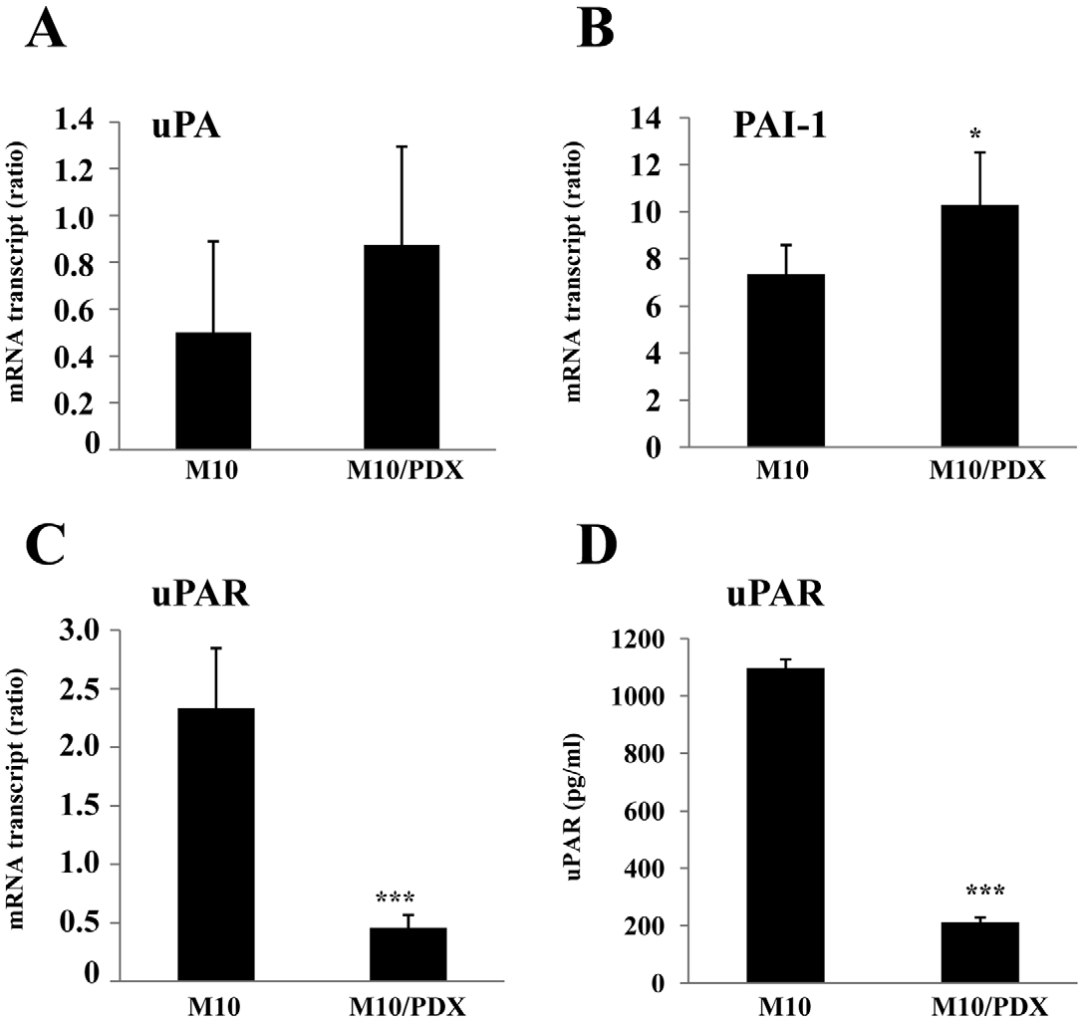
Effect of PCs inhibition on uPA, uPAR and PAI-1 expression. (**A–C**), Following total RNA extraction from indicated cells, real-time PCR analysis was performed using specific primers for indicated genes as described in Materials and Methods. Results are shown in the bar graph and are expressed as the mRNA transcript ratios. Data are shown as means ± S.E of three experiments performed in duplicate. Student's *t* test was used for statistical analysis. *, *P*<0.05 and ***, *P*<0.001. (**D**), Tumor cells were incubated at 37°C in serum-free media. After 24 hours, media were collected and analyzed for the presence of uPAR using ELISA kit. Results shown are representative of 3 experiments. Data are mean ± SEM (*n = *6 per group). Student's *t* test was used for statistical analysis ***, *P*<0.001

Similarly, since the uPA/uPAR/PAI-1 system is also involved in melanoma cell invasion [35], we analyzed the effect of PCs inhibition on the expression levels of these molecules by real-time PCR and ELISA assays in M10 and M10/PDX. Fig. 6 shows that the level of uPA did not change significantly (Fig. 6A), PAI-1 was increased (Fig. 6B), whereas the level of uPAR mRNA was dramatically decreased (Fig. 6C). Similarly, at the protein level, ELISA assay revealed that the amount of uPAR collected 24 hours following incubation of cells in serum-free media was decreased significantly in cells expressing α1-PDX (Fig. 6D).

## Discussion

The loss of function of tumor repressor genes *p53* and/or *CDKN2A* contributes to the induction and/or over-expression of various extracellular matrix-degrading enzymes including MMPs and urokinase, leading to increased tumor cells invasiveness [6], [8]–[12]. Accordingly, the restoration of *p53* and/or *CDKN2A* expressions was found to inhibit MMPs expression and tumor cells invasion [6], [8]–[12]. In this study, we found that inhibition of activity of proprotein convertases (PCs) in human primary melanoma cells with altered *p53, CDKN2A* and *Ras* genes, provides a potent repression mechanism of primary melanoma cell invasiveness by interfering with the expression and/or activity of several extracellular matrix degrading enzymes and their inhibitors.

**Figure 7:**
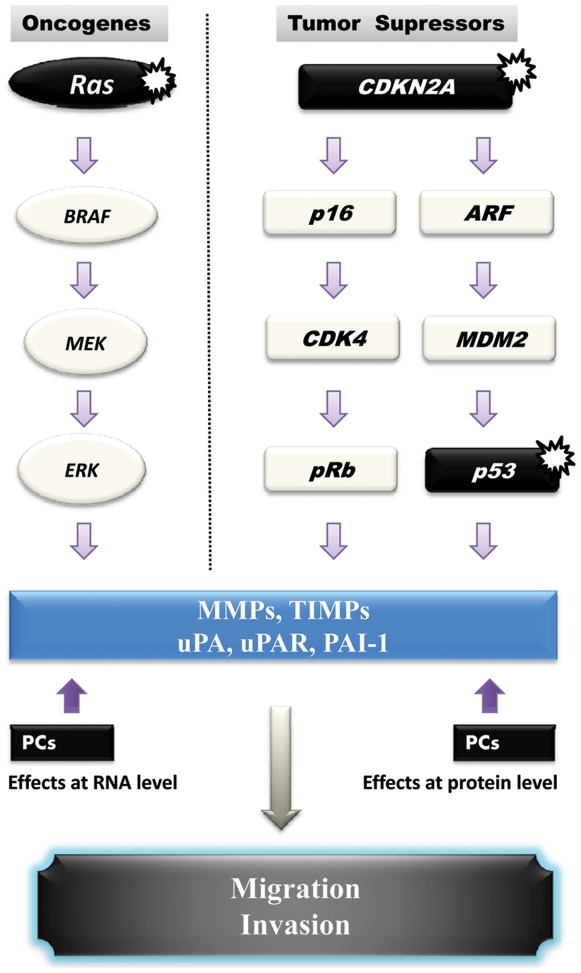
Schematic representation of the effects of PCs inhibition on migration and invasion of human melanoma cells with altered *CDKN2A*, *p53* and *Ras* genes. The *CDKN2A* locus codes for the tumor suppressors p16 and ARF that regulate cell cycle progression, DNA repair and cell invasion via activating pRb, and the p53 pathway via inhibiting the activity of MDM2, respectively. Mutations or deletions of the *CDKN2A* gene result in altered MMPs/TIMPs and/or uPA/uPAR/PAI-1 expression and activity, which in turn lead to tumor cell invasion. The *Ras* gene is important for regulating the ERK activity, and hence expression of MMPs and uPA/uPAR. An activating mutation in the Ras gene can result in uncontrolled activity of MMPs and the urokinase system, which can lead to increased melanoma invasion. Blockade of PCs activity can reduce the expression and/or generation of active MMPs, uPA and uPAR leading to reduced tumor cells invasiveness. The asterisks indicate altered genes.

It is well established that invasiveness and the ability of cancer cells to migrate *in vitro* and *in vivo* is mediated by various metalloproteases (including MMP-2 and MMP-9) and urokinase plasminogen system (uPA, uPAR and PAI-1) [36]. Indeed, data reported on the activity of MMPs in different cells and tissues, indicate that various MMPs are capable of degrading most components of the basement membrane and ECM, facilitating tumor cell migration and invasion [35]–[37]. The proteolytic activity of these MMPs is tightly regulated by their inhibitors TIMPs. Thereby the equilibrium between activation and inhibition that affect the MMPs and their inhibitors leads to excessive proteolysis or inhibition [35]–[37]. The regulation of invasion by the convertases was previously reported in numerous studies. It was demonstrated that convertases directly involved in the activation of several MMPs ( MT-MMPs), or indirectly, as in the case of MMP-2 [22]. Similarly, the binding of uPA to its receptor uPAR induces its activation that in turn activates plasminogen and generates the active plasmin involved in the degradation of ECM [37]. These processes were previously reported to promote tumor invasion and migration [37]. Thereby targeting these proteases and their activators inhibits the migration and invasion of tumor cells. To investigate whether inhibition of PCs in primary melanoma cells represses the malignant phenotype of tumor cells with altered *p53* and *CDKN2A* tumor repressor genes, we used primary human melanoma M10 cells that we found to have deleted *CDKN2A* and mutated *p53* (Fig. 1). Analysis of corresponding gastric and lymph node metastatic lesions, MT10 and MG10 cells, revealed the presence of identical gene alterations. Following stable expression of the general PCs inhibitor in M10 cells, activity of PCs was repressed, as revealed by in *vitro* enzymatic digestion assay, as well as by their inability to process PCs substrates pro-PDGF-A and pro-IGF-1R (Fig. 2C, 2D). Indeed, these tumor cells expressed all the PCs found in the secretory pathway namely, Furin, PACE4, PC5 and PC7 (Fig. 2A, 2B). The inhibition of their PCs activity by α1-PDX inhibited their migration and invasion (Fig. 3 and Fig. 4) that was associated with reduced MMP-2 activity and increased level of TIMP-1 and TIMP-2 (Fig. 5). These data are in agreement with a previous report showing that over-expression of Furin in tumor cells results in increased MMP-2 activity and cell invasion [22]. In these studies, the increased MMP-2 activity in tumor cells expressing Furin was linked to the increased activity of MT1-MMP, which is processed by Furin [38]. In other systems, expression of α1-PDX in tumor cells blocked the maturation of MT1-MMP, leading to reduced MMP-2 activity and cell invasion [22]. In the current study, we revealed that the PCs can also control the activity of MMP-2 by acting on the expression levels of MMP-2 inhibitors TIMP-1 and TIMP-2 (Fig. 5) and the levels of uPAR and PAI-1 in tumor cells (Fig. 6). However, the inhibitory effect of α1-PDX on MT1-MMP processing and MMP-2 activity leading to reduced cell migration and invasion has not been ruled out in our model. The critical role of the PCs in the proteolytic maturation of multiple precursor substrates implicated in neoplasia makes these enzymes attractive targets in cancer therapy [39]. Indeed, introducing mutations in several PCs substrates expressed in various tumor cells resulted in reduced invasiveness and tumorigenicity in nude mice [22]-[26], [38]. Accordingly, over-expression of Furin has been established in various types of human cancers and has been linked to aggressive behavior of tumor cells [22], [23]. These changes were directly related to the inhibition of Furin-mediated activation of several cancer-related substrates [22], [28]. Therefore, the observed reduced activity of MMP-2 in M10/PDX cells suggests that inhibiting the PCs could alter the invasive and metastatic phenotypes of melanoma cells induced by altered *p53, CDKN2A* genes and/or probably other genes such as *Ras* (Fig. 7). It is likely that α1-PDX can block the processing of other proteins implicated in the invasive potential of tumor cells, such as growth factors and adhesion molecules, as previously reported *in vitro* and *in vivo* models [22], [23], [25], [38].

In conclusion, our results support the hypothesis that the malignant phenotype of primary human melanoma with altered tumor repressor genes *p53* and *CDKN2A* can be abrogated by inhibition of PCs activity (Fig. 7). These enzymes may thus constitute novel therapeutic targets for melanoma treatment, leading to the development and application of potent bio-available PCs inhibitors.
